# Association of whole grain, refined grain, and cereal consumption with gastric cancer risk: A meta‐analysis of observational studies

**DOI:** 10.1002/fsn3.878

**Published:** 2018-11-11

**Authors:** Yujie Xu, Jie Yang, Liang Du, Ka Li, Yong Zhou

**Affiliations:** ^1^ West China School of Nursing and Department of Nursing West China Hospital Sichuan University Chengdu China; ^2^ Department of Gastrointestinal Surgery West China Hospital Sichuan University Chengdu China; ^3^ Chinese Evidence‐Based Medicine/Cochrane Center Chengdu China; ^4^ Department of Nursing West China Hospital Sichuan University Chengdu China

**Keywords:** cereal, gastric cancer, meta‐analysis, refined grain, whole grain

## Abstract

Many studies have analyzed the relationship between cereal, whole, or refined grain and the risk of gastric cancer (GC) and have yielded mixed results. Therefore, we performed a meta‐analysis of observational studies to summarize the available evidence on this topic. Databases such as PubMed, EMBASE, Web of Science, MEDLINE, and the Cochrane Library were searched for studies focusing on these associations from inception to October 2017. Summary odd ratios (OR) and 95% confidence intervals (CI) were calculated by using either a random‐ or fixed‐effect model according to the between‐studies heterogeneity. Subgroup analysis was also performed. In total, eleven studies that included 530,176 participants were identified. In a pooled analysis of all studies, cereal exposure was not associated with GC risk (OR, 1.11, 95%CI, 0.85–1.36). Specific analyses indicated that whole grain consumption was associated with decreased GC risk (OR, 0.61, 95%CI, 0.40–0.83) and that refined grain consumption was associated with increased GC risk (OR, 1.65, 95%CI, 1.36–1.94). Higher whole grain and lower refined grain intake but not cereal consumption reduces GC risk. This study has been registered at ClinicalTrials.gov (ID: NCT03419663).

## 1 INTRODUCTION

Globally, gastric cancer (GC) is the fifth most common malignancy, and its prevalence varies among countries as well (Ferlay et al., 2015; Torre et al., 2015). In general, more than 70% of cases occur in developing countries and account for half of the total cases in Eastern Asia, most of which occur in China (Colquhoun et al., 2015). Moreover, GC is the third major cause of cancer death worldwide (Ferlay et al., 2015), although its incidence and mortality rates have been steadily decreased in most parts of the world (Bertuccio et al., 2009; Malvezzi et al., 2010). Hence, there is an urgent demand for effective prevention to reduce the risk of GC (Lansdorp‐Vogelaar & Kuipers, 2016). The recognition of genetic and environmental risk factors in the development of GC has gained increasing attention and might contribute to its prevention by a series of preventative measures. To date, three key risk factors of unhealthy lifestyle on GC are smoking, poor diet, and infection caused by *Helicobacter pylori* (El‐Serag & Sonnenberg, 1999; Larsson, Orsini, & Wolk, 2006; Plummer, Franceschi, Vignat, Forman, & de Martel, 2015; Steevens, Schouten, Goldbohm, & van den Brandt, 2010; Wong et al., 2004). Among known dietary factors, alcohol use (Rota et al., 2017) and processed meat intake (Larsson et al., 2006) are widely accepted to be related to an increased GC risk, while fresh vegetables or fruits (Lunet et al., 2007), allium vegetables (Zhou et al., 2011), and dietary fiber (Zhang, Xu, Ma, Yang, & Liu, 2013) have inverse associations with GC occurrence. Meanwhile, other factors need further investigation.

Cereal and grains are the main staple foods in majority of the world, providing most of the energy in diets, and contain 70%–72% starch, 7%–15% protein, 1%–2% lipid, and water (Sturgess, Ellis, & Ciclitira, 1991). In lower income countries, total population consumption of these foods may amount to 60%–80% of total energy (Research, W. C. R. F. A. I. f. C., 2007). A cereal is any grass cultivated for the edible components of its grain, while a grain is a small, hard, dry seed, with or without an attached shell layer, and both are composed of the endosperm, germ, and bran (Tetens, 2017). Many epidemiological studies have investigated the relationship between cereal and grain with GC risk, but the results have not been consistent. In 1967, Hakama drew the conclusion that cereal consumption increased the incidence of GC, on the basis of the discovery of low rates of such cancer among the citizens worldwide who ate a diet with a decreased proportion of cereal (Hakama & Saxen, 1967). However, the correlation observed may be indirect and connected to other unknown factors. In 1997, a pooled analysis investigating the association between dietary factors and GC found that cereal and other high‐starch foods were postulated to increase GC, but the association was not significant (Kono & Hirohata, 1996). Moreover, the effect of specific types of cereal or grain on GC risk was not known with any certainty. Whole grain was reported to have an inverse association with GC risk (Aune et al., 2016; Chatenoud et al., 1998), but refined grain was mostly positively associated with the risk (Chatenoud et al., 1999).

Cereal consumption, especially whole grain, has been evaluated the risk of colorectal cancer (Aune et al., 2011). However, to the best of our knowledge, no systematic review or meta‐analysis of observational studies has been conducted that focus on GC risk and the use of cereal or grain. This meta‐analysis aims to investigate the role of cereal and grain in GC risk and examine in depth the function of specific types of cereal and grain, including whole grains, refined grains, and other grains.

## 2 MATERIALS AND METHODS

This study has been registered at ClinicalTrials.gov (ID: NCT03419663).

### 2.1 Search strategy

We searched PubMed, EMBASE, Web of Science, MEDLINE, and Cochrane Library through October 2017 for studies reporting the association between cereal or grain consumption and the risk of GC, with no restrictions on language. The following three themes of medical terms were used in combination: (cereal OR grain) AND (gastric OR stomach) AND (cancer OR neoplasm OR tumor OR malignancy OR carcinoma OR adenocarcinoma). The reference lists of included articles and published systematic reviews or meta‐analyses were carefully assessed to identify additional studies as well. Moreover, we contacted first authors to ask for additional information when necessary.

### 2.2 Study selection and data extraction

The criteria for study selection were as follows: (a) a case–control study or cohort study; (b) investigation of the association between cereal or grain intake and GC occurrence; (c) the odds ratio (OR) or relative risk (RR) with confidence intervals (CI) or data necessary to estimate these statistics were provided. We excluded GC mortality‐based cohort studies. If a study was reported multiple publications, we generally selected the most recent one with the largest sample size.

Eligible studies were independently evaluated by two authors (Yujie Xu and Yong Zhou), and a third author (Ka Li) resolved any discrepancies. For each study, the following data were extracted into data collection form: abbreviation of the main researcher's name, publication year, study design, participants’ nationalities, follow‐up duration, numbers of cases (patients diagnosed with GC) and participants, categories of cereal consumption, quantities of cereal intake in each category, adjusted OR or RR with corresponding 95%CI for the highest versus the lowest intake as well as across more than two exposure levels in each category, and adjustment factors.

### 2.3 Statistical analysis

The PRISMA checklist was used as a protocol and guideline of this meta‐analysis. We investigated the association between cereal intake and GC risk based on the effect estimates and their 95%CI in each included report. The OR mathematically approximates the RR in case–control studies owing to the low rates of GC; therefore, the measure of effect is OR along with 95%CI in our meta‐analysis. For the pooled analysis, we used either fixed‐effect models when heterogeneity was low or random‐effect models that take heterogeneity within and between studies into account to calculate the summary OR.

The Cochran's Q test and *I* ^2^ statistics were used to assess statistical heterogeneity across studies. A *p* value <0.10 for the *Q* test or *I* ^2^ >50% was considered to indicate heterogeneity. Subgroup analysis according to study design and cereal consumption categories was also conducted to evaluate the potential source of heterogeneity.

The presence of publication bias was investigated by using the Begg's and Egger's tests, and the results were considered to indicate potential bias at *p *< 0.05. We also carried out a traditional sensitivity analysis and excluded a study with extreme data to explore whether the results were stable or driven by a specific study. All quantitative analyses were conducted by using Stata software (version 12.0; StataSE).

## 3 RESULTS

### 3.1 Literature search

**Figure 1:**
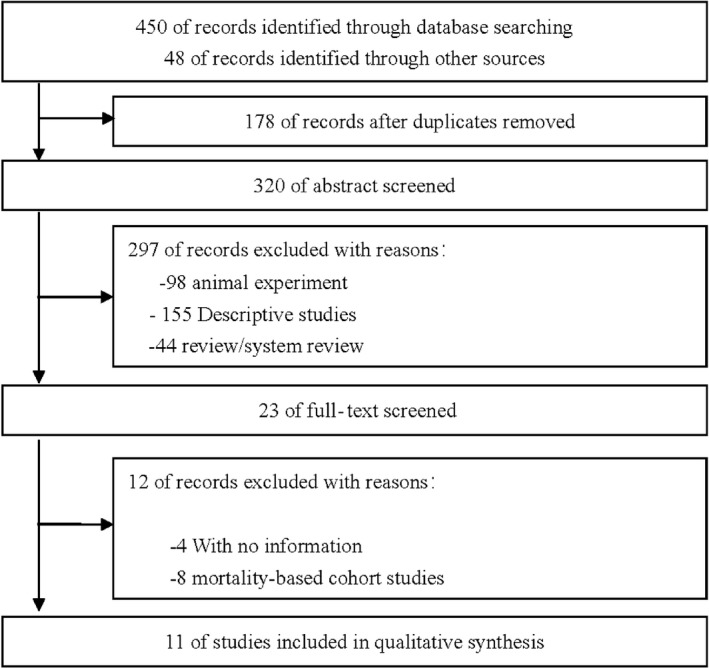
Flowchart of article selection

Figure 1 presents the process of study screening and selection. Overall, the search yielded 498 articles, and 320 remained for further evaluation after 178 duplicated articles were excluded. Of the 23 full‐text reports remaining after title and abstract screening, four studies (Denova‐Gutierrez, Hernandez‐Ramirez, & Lopez‐Carrillo, 2014; Karagulle, Fidan, Kavgaci, & Ozdemir, 2014; Terry, Lagergren, Ye, Wolk, & Nyren, 2001; Wang et al., 2016) without available OR and 95% CI were ruled out, and another eight studies (Fraser, 1999; Gil, Ortega, & Maldonado, 2011; Jansen et al., 1999; Lafiandra, Riccardi, & Shewry, 2014; McCullough et al., 2001; Shamberger, Tytko, & Willis, 1972; So, Law, Law, Chan, & Chair, 2016; Vanamala, Massey, Pinnamaneni, Reddivari, & Reardon, 2017) were not included because they did not investigate cereal or grain consumption and GC risk. After this review, 11 observational studies evaluating the risk of GC remained for the analysis (Buckland et al., 2010; Chatenoud et al., 1999; Chen et al., 2002; De Stefani et al., 2004; Kasum, Jacobs, Nicodemus, & Folsom, 2002; Lissowska et al., 2004; Lucenteforte et al., 2008; Ramón, Serra, Cerdó, & Oromí, 1993; Wang et al., 2012; Ward, 1999; Zhang et al., 1997). A manual search of the reference lists did not increase the number of included articles.

### 3.2 Study characteristics

A summary of study characteristics of individual studies is presented in Table 1. The included studies comprised 527,256 individuals and 2,920 GC cases and were published from 1993 to 2012. Of 11 studies, five (Chatenoud et al., 1999; Lucenteforte et al., 2008; Ramón et al., 1993; Wang et al., 2012; Zhang et al., 1997) were hospital‐based case–control studies, four (Chen et al., 2002; De Stefani et al., 2004; Lissowska et al., 2004; Ward, 1999) were population‐based case–control studies, two (Buckland et al., 2010; Kasum et al., 2002) were prospective cohort studies. Among these, three studies were performed in the United States, two in Italy, and each one in China, Spain, Poland, Mexico, and Uruguay. One study included participants from multiple European countries. The adjustment factors in these studies included age, sex, education, total intake of calories, pack‐years of smoking, alcohol use, body mass index (BMI), family history of GC, intake of fruits or vegetables.

**Table 1:** Characteristics of studies of cereal consumption and gastric cancer risk

Study (year)	Design	Study period	Country	Cases/Controls, n	Type of cereal and consumption categories	OR/RR (95%CI)	Adjustment
Chatenoud et al.,^1999^);	HCC	1983–1993	Italy	745/3526	Refined cereals (portions/week)		Center, age, sex, education, smoking habits, alcohol intake, BMI, intake of fruit vegetable
					≤14	1.0	
					15–21	1.24 (1.0–1.5)	
					≥22	1.54 (1.2–2.0)	
					Whole grains	0.5 (0.4–0.7)	
Ramón et al.,^1993^);	HCC	1986–1989	Spain	117/234	Cereals (g/d)		Sex, age, education, and cigarettes/day.
					<291.8	1.0	
					291.8–338.2	1.02 (0.67–2.01)	
					338.2–410.6	1.31 (0.87–2.29)	
					>410.6	1.83 (0.65–4.01)	
Zhang et al.,^1997^);	HCC	1992–1994	United States	95/132	Grains and Cereals		Age, sex, race, education, total dietary intake of calories, pack‐years of smoking, alcohol use, and body mass index
					1 (low)	0.6 (0.2–1.7)	
					2	0.7 (0.3–2.0)	
					3	0.6 (0.2–1.4)	
					4 (high)	0.8 (0.5–1.1)	
Ward,^1999^);	PCC	1989–1990	Mexico	220/752	Grains and Cereals (times/day)		Age, gender, total calories, chili pepper consumption, added salt, history of peptic ulcer, cigarette smoking, and socioeconomic status
					<3	1.0	
					3	0.8 (0.4–1.4)	
					4–5	0.9 (0.5–1.6)	
					≥6	0.7 (0.4–1.6)	
Lucenteforte et al.,^2008^);	HCC	1997–2007	Italy	230/547	Cereals (servings/wk)		Sex, age, education, year of interview, BMI, tobacco smoking, family history of stomach cancer, total energy intake
					1 (low)	1.0	
					2	1.29 (0.66–2.52)	
					3	1.57 (0.81–3.07)	
					4	1.69 (0.86–3.35)	
					5 (high)	2.07 (1.01–4.24)	
Lissowska et al., 2004;	PCC	1994–1996	Poland	274/463	Grains total		Age, sex, education, smoking, calories from food
					1 (low)	1.0	
					2	1.37 (0.71–1.57)	
					3	1.58 (0.49–1.15)	
					4 (high)	1.89 (1.00–2.85)	
					Refined grains		
					1 (low)	1.0	
					2	1.50 (0.94–2.39)	
					3	1.70 (1.03–2.81)	
					4 (high)	1.80 (1.04–3.13)	
					Whole grains		
					1 (low)	1.0	
					2	1.01 (0.65–1.57)	
					3	1.32 (0.86–1.04)	
					4 (high)	1.05 (0.65–1.69)	
De Stefani et al.,^2004^);	PCC	1996–2000	Uruguay	240/960	Grains		Age (categorical), sex, residence, urban/rural status, education (categorical), body mass index (categorical), and total energy intake
					1 (low)	1.0	
					2	1.63(1.07–2.49)	
					3 (high)	1.83 (1.17–2.85)	
Kasum et al.,^2002^);	Cohort study	1986–1999	Iowa	169/34,651	Whole grains (servings/week)		Age, pack‐year of smoking, alcohol use, and energy intake
					0–6.5	1.0	
					6.9–12.5	1.01 (0.67–1.35)	
					13.0–108.5	0.61 (0.34–0.81)	
					Refined grains (servings/week)		
					0–4.0	1.0	
					4.5–9.0	1.73 (1.39–2.31)	
					9.5–78.0	1.76 (1.42–2.35)	
Chen et al.,^2002^);	PCC	1992–1994	United States	124/449	Cereals		Age, sex, energy intake, respondent type, BMI, alcohol use, tobacco use, education, family history, vitamin supplement use
					1 (low)	1.0	
					2	1.2 (0.62–2.4)	
					3	0.74 (0.35–1.5)	
					4 (high)	0.71 (0.32–1.6)	
Buckland et al.,^2010^);	Cohort study	1992–1998	Europe	449/485,028	Cereals (g/Kcal·d)		Age and adjusted for sex (in overall model), BMI, educational level, smoking status, cigarette smoking intensity, and total energy intake.
					1 (low)	1.0	
					2	0.99 (0.79–1.26)	
					3 (high)	0.80 (0.61–1.04)	
Wang et al.,^2012^)	HCC	2008–2010	China	257/514	Cereals		Education, smoking, alcohol consumption, family history, total vegetable intake, total fruit intake, pickled food, Soy products, total energy intake, meat, and H pylori.
					1	1.0	
					2	1.4 (0.5–3.5)	
					3	1.5 (0.8–3.2)	

BMI, body mass index; CI, confidence interval; HCC, hospital‐based case–control; OR, odds ratio; PCC, population‐based case–control.

### 3.3 Main analysis

**Figure 2:**
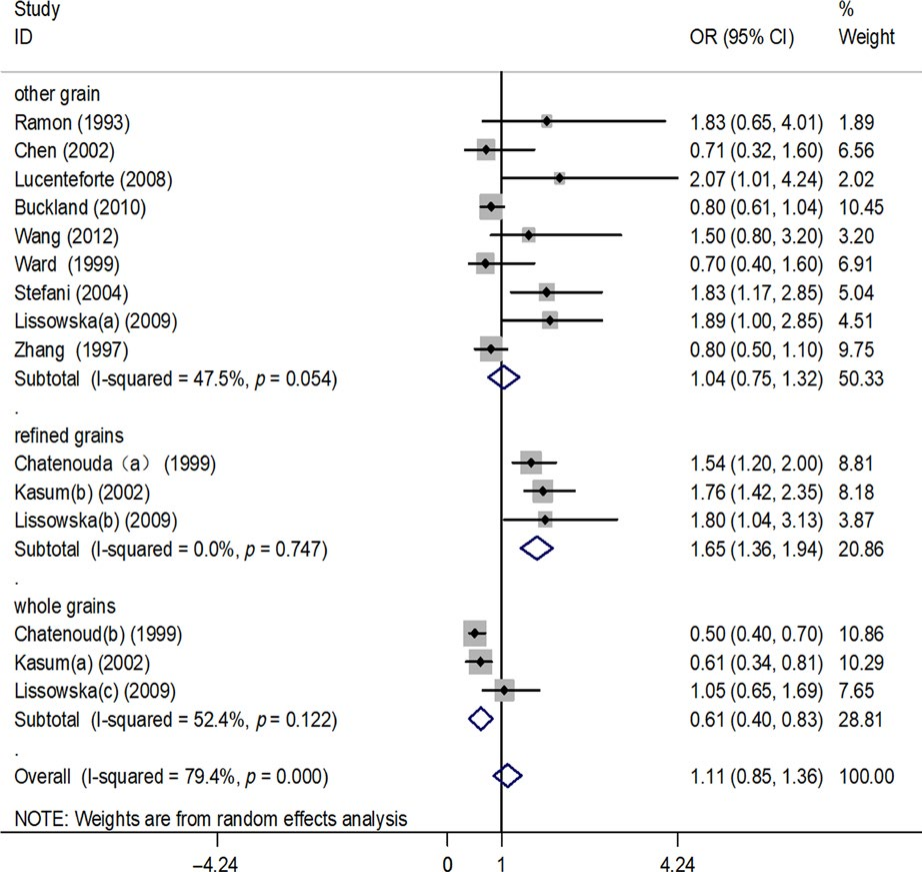
Forest plot (random‐effects model) of cereal or grain consumption (highest versus lowest category) and gastric cancer risk

For the analyses of the relation of GC with cereal or grain consumption, we included nine case–control studies and two cohort studies presenting data on the issue. In the pooled analysis of all studies, cereal exposure was not associated with risk of GC (OR, 1.11, 95%CI, 0.85–1.36). Specific analyses for cereal, whole grains, refined grains, and other grains were also conducted. Among the two case–control studies and one cohort study assessing whole grain or refined grain consumption, higher whole grain intake was related to a statistically significant decrease in GC morbidity risk (OR, 0.61, 95%CI, 0.40–0.83), while a higher category of refined grain intake increased the incidence of GC (OR, 1.65, 95%CI, 1.36–1.94). In addition, the meta‐analysis revealed substantial heterogeneity across studies with low intake of whole grains (*I* ^2^ = 52.4%, *p* for heterogeneity = 0.12) and refined grain (*I* ^2^ = 0.00%, *p* for heterogeneity = 0.75). For intake of other grains, which was not significantly associated with GC risk, the pooled OR was 1.04 (95%CI, 0.75–1.33), and heterogeneity was significant (*I* ^2^ = 47.5%, *p* for heterogeneity = 0.05). The results of main analysis and specific analyses are reported in Table 2 and Figure 2.

**Table 2:** Meta‐analysis of cereal consumption and gastric cancer risk

Type of cereals	No. of participants	Random	Fixed	*P*_heterogeneity_
		OR (95%CI)	OR (95%CI)	
Whole grain	39,640	0.61 (0.40,0.83)	0.56 (0.44,0.68)	0.12
Refined grain	39,640	1.65 (1.36,1.94)	1.65 (1.36,1.94)	0.75
Other grain	489,049	1.04 (0.75,1.32)	0.89 (0.73,1.04)	0.05
Total cereals	530,176	1.11 (0.85,1.36)	0.79 (0.69,0.89)	<0.001

CI, confidence interval; OR, odds ratio.

**Figure 3:**
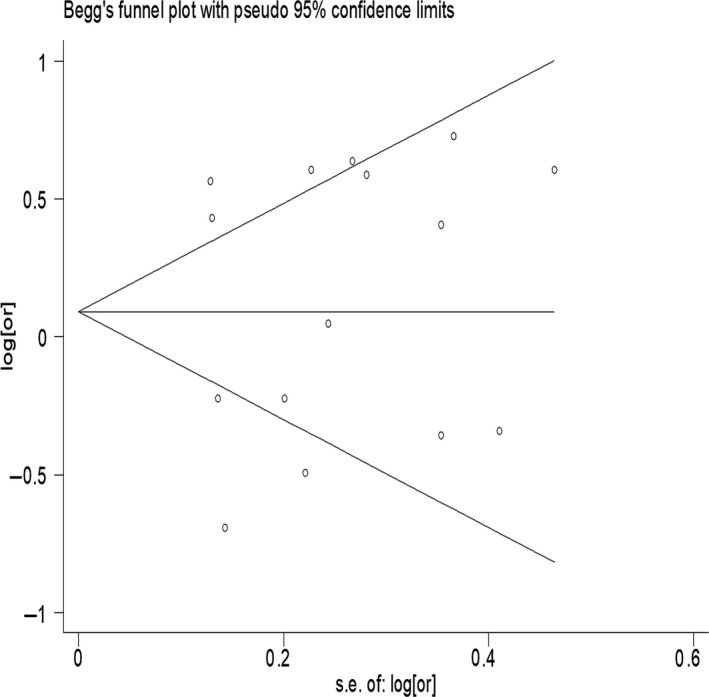
Publication bias of all studies by Begg's test

**Figure 4:**
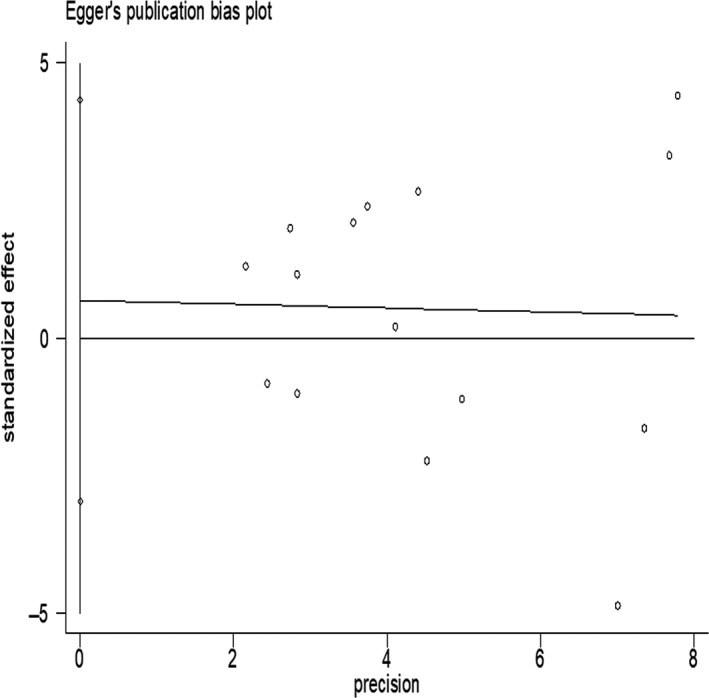
Publication bias of all studies by Egger's test

### 3.4 Subgroup analysis

The summary OR for the association of whole grains, refined grains, and other grains with GC risk stratified by selected variables is presented in Table 3. For stratification by geographic area, the relationship of whole grain with GC risk was statistically significant for studies performed in America (OR, 0.61, 95%CI, 0.38–0.85), but not for those performed in Europe (OR, 0.72, 95%CI, 0.19–1.24). For refined grain consumption, the ORs were 1.58 (95%CI, 1.20–1.95) in Europe and 1.76 (95%CI, 1.30–2.23) in America. For stratification by study design, a significant association was observed for whole grains in hospital‐based case–control (OR, 0.50, 95%CI, 0.35–0.65) and cohort (OR, 0.61, 95%CI, 0.38–0.85) studies but not in population‐based case–control (OR, 1.05, 95%CI, 0.53–1.57) studies. The corresponding value for refined grain was 1.54 (95%CI, 1.14–1.94) in hospital‐based case–control studies, 1.76 (95%CI, 1.30–2.23) in cohort studies, and 1.05 (95%CI, 0.53–1.57) in population‐based case–control studies. However, the association between other grain exposure and GC risk was not statistically significant in the subgroup analysis according to study design or geographic area.

**Table 3:** Subgroup analysis of cereal consumption and gastric cancer risk

Subgroup	Whole grain	Refined grain	Other grain
	OR (95%CI)	OR (95%CI)	OR (95%CI)
Geographic area			
Europe^a^	0.72 (0.19, 1.24)	1.58 (1.20, 1.95)	0.89 (0.69, 1.09)
America^b^	0.61 (0.38, 0.85)	1.76 (1.30, 2.23)	0.85 (0.62, 1.10)
Asia^c^	NA	NA	1.5 (0.3, 2.7)
Study design			
HCC	0.50 (0.35, 0.65)	1.54 (1.14, 1.94)	0.91 (0.62, 1.19)
PCC	1.05 (0.53, 1.57)	1.80 (0.76, 2.85)	1.09 (0.73, 1.44)
Cohort	0.61 (0.38, 0.85)	1.76 (1.30, 2.23)	0.80 (0.59, 1.01)

CI, confidence interval; HCC, hospital‐based case–control; NA, not applicable; OR, odds ratio; PCC, population‐based case–control. Europe^a^ includes Spain, Italy, Poland, and other European countries; America^b^ includes United States, Mexico, Uruguay; Asia^c^ includes China.

### 3.5 Sensitivity analyses and publication bias

Sensitivity analyses were further carried out, excluding a study with extreme data, and the results obtained were essentially unchanged compared with those of the main analysis. Begg's and Egger's tests (Figures 3 and 4) were used to test publication bias between studies and did not indicate significant bias (Begg's test, *p *= 0.84; Egger's test, *p *= 0.69).

## 4 DISCUSSION

We meta‐analyzed the evidence regarding the relationship between cereal or grain consumption and the risk of GC. Our meta‐analysis indicated that higher whole grain intake was associated with lower GC risk, higher refined grain intake was associated with higher risk of GC, and no association was found between overall cereal and GC risk.

A previous meta‐analysis (Chatenoud et al., 1998) showed an inverse association between whole grain consumption and GC incidence; however, this association was based on a few studies conducted only in Italy where whole grain food consumption was small. Furthermore, their study was not population‐based, and GC cases were from major hospitals. Another meta‐analysis (Jacobs, Marquart, Slavin, & Kushi, 1998) included case–control studies indicating that whole grain intake was inversely related to all digestive tract cancers, including GC, but some of the included studies typically focused on whole grain fiber, rather than separated whole grain products in the diet. For refined grain consumption, there are some original studies showing that diets rich in starches and refined grains were associated with an increased risk of GC (Buiatti et al., 1989; La Vecchia, Negri, Decarli, D'Avanzo, & Franceschi, 1987; Modan et al., 1974), but the results were inconsistent. Therefore, we performed this meta‐analysis of case–control studies or cohort studies conducted all over the world to evaluate the relationship of overall cereal, whole grain, refined grain, and other grain consumption with GC risk and to compare these association between subgroups.

In our meta‐analysis, as in previous reports, higher whole grain intake and lower refined grain intake may decrease GC risk. The reasons for this finding could be as follows: (a) whole grain is made up of germ, endosperm, and bran, and the latter two contain a large amount of nutrients. Refined grain includes only the endosperm. Moreover, whole grain is a major source of dietary fiber, vitamins, and several minerals (Stephen, 1994). (b) The whole grain diet, compared with a refined grain diet, plays a role in decreasing energy intake and lowering systemic inflammation without significantly altering gut microbiome or gut functionality in terms of intestinal integrity and transit time (Costabile et al., 2008; Roager et al., 2017). (c) Refined grain has a higher digestibility than whole grain, resulting in glycemic overload, higher plasma insulin concentration and compensatory increases in insulin‐like growth factor I, an important mitogenic stimulant of tumor cell growth in vitro (Giovannucci, 1995). With the two aspects of cereal or grain products, overall cereal intake showed a nonsignificant association with GC risk.

Interestingly, studies have confirmed that whole grain intake had an inverse association with other cancers. Levi et al. (2000) conducted a meta‐analysis and found that whole grain was a favorable indicator of the risk of upper digestive (oral, esophageal) and respiratory tract (laryngeal) neoplasms, while refined grain was an unfavorable predictor. A prospective study performed by Schatzkin showed an inverse association between whole grain foods and small intestinal cancer (Schatzkin, Park, Leitzmann, Hollenbeck, & Cross, 2008). Higher whole grain consumption is widely accepted to be able to reduce colorectal cancer risk (Aune et al., 2011; Kyro et al., 2014; Larsson, Giovannucci, Bergkvist, & Wolk, 2005; Schatzkin et al., 2007). Moreover, another meta‐analysis of observational studies suggested that whole grain intake was associated with a decreased risk of pancreatic cancer (Lei et al., 2016). However, there was no association between whole grain consumption and prostate cancer (Egeberg et al., 2011), breast cancer among postmenopausal women (Egeberg et al., 2009), and endometrial cancer (Kasum, Nicodemus, Harnack, Jacobs, & Folsom, 2001). Therefore, more prospective studies are needed to investigate the role of whole grain in health.

To our knowledge, this was the first meta‐analysis performed to summarize the effect of cereal intake on GC risk. First, the extensive search strategy was conducted to identify all relevant global publications. Second, another strength of this study was the large number of participants from Asia, America, and Europe, allowing further subgroup analyses. Third, although different types of studies, such as case–control and cohort studies, were included, between‐study heterogeneity was small, and publication bias was not found.

There are also several limitations in our study. First, we found no data on some tumor types (such as cardia GC or noncardia GC) that deserve attention by clinical researchers. Therefore, we need more investigation into the association of cereals with the risk of different types of GC. Second, most of the studies in this meta‐analysis were case–control studies. Third, the number of studies for the whole grain and refined grain analyses was small. Finally, although all included studies were adjusted for potential confounding factors, we cannot exclude the possibility that some other unmeasured factors might be responsible for the inverse relationship.

In conclusion, intake of higher whole grains and lower refined grains but not cereal reduces GC risk. Our findings may provide useful evidence for public health researchers and help provide a proposal for appropriate cereal food consumption among healthy citizens in the near future. However, further well‐designed studies are still needed.

## CONFLICT OF INTEREST

The authors declare that they do not have any conflict of interest.

## AUTHOR CONTRIBUTIONS

The authors’ responsibilities were as follows—YJX, YZ, and KL: designed the study; YZ and YJX: conducted the library search and wrote the manuscript; YZ and JY: extracted and controlled the data; YJX and LD: analyzed the data; YZ and KL: had primary responsibility for the final content; and all authors: read and approved the final manuscript.

## ETHICAL REVIEW AND INFORMED CONSENT

This study does not involve any human or animal testing, and all analyses are based on previous published studies, thus no ethical review and patient consent are required.

## References

[fsn3878-bib-0001] Aune, D. , Chan, D. S. , Lau, R. , Vieira, R. , Greenwood, D. C. , Kampman, E. , & Norat, T. (2011). Dietary fibre, whole grains, and risk of colorectal cancer: Systematic review and dose‐response meta‐analysis of prospective studies. BMJ, 343, d6617 10.1136/bmj.d6617 22074852 PMC3213242

[fsn3878-bib-0002] Aune, D. , Keum, N. , Giovannucci, E. , Fadnes, L. T. , Boffetta, P. , Greenwood, D. C. , … Norat, T. (2016). Whole grain consumption and risk of cardiovascular disease, cancer, and all cause and cause specific mortality: Systematic review and dose‐response meta‐analysis of prospective studies. BMJ, 353, i2716 10.1136/bmj.i2716 27301975 PMC4908315

[fsn3878-bib-0003] Bertuccio, P. , Chatenoud, L. , Levi, F. , Praud, D. , Ferlay, J. , Negri, E. , … La Vecchia, C. (2009). Recent patterns in gastric cancer: A global overview. International Journal of Cancer, 125, 666–673. 10.1002/ijc.24290 19382179

[fsn3878-bib-0004] Buckland, G. , Agudo, A. , Lujan, L. , Jakszyn, P. , Bueno‐de‐Mesquita, H. B. , Palli, D. , … González, C. A. (2010). Adherence to a Mediterranean diet and risk of gastric adenocarcinoma within the European Prospective Investigation into Cancer and Nutrition (EPIC) cohort study. The American Journal of Clinical Nutrition, 91, 381–390. 10.3945/ajcn.2009.28209 20007304

[fsn3878-bib-0005] Buiatti, E. , Palli, D. , Decarli, A. , Amadori, D. , Avellini, C. , Bianchi, S. , Biserni, R. , Cipriani, F. , Cocco, P. , Giacosa, A. , … Blot, W. (1989). A case‐control study of gastric cancer and diet in Italy. International Journal of Cancer, 44, 611–616. 10.1002/(ISSN)1097-0215 2793233

[fsn3878-bib-0006] Chatenoud, L. , La Vecchia, C. , Franceschi, S. , Tavani, A. , Jacobs, D. R. Jr , Parpinel, M. T. , … Negri, E. (1999). Refined‐cereal intake and risk of selected cancers in Italy. The American Journal of Clinical Nutrition, 70, 1107–1110. 10.1093/ajcn/70.6.1107 10584057

[fsn3878-bib-0007] Chatenoud, L. , Tavani, A. , La Vecchia, C. , Jacobs, D. R. Jr , Negri, E. , Levi, F. , & Franceschi, S. (1998). Whole grain food intake and cancer risk. International Journal of Cancer, 77, 24–28. 10.1002/(ISSN)1097-0215 9639389

[fsn3878-bib-0008] Chen, H. , Ward, M. H. , Graubard, B. I. , Heineman, E. F. , Markin, R. M. , Potischman, N. A. , … Tucker, K. L. (2002). Dietary patterns and adenocarcinoma of the esophagus and distal stomach. The American Journal of Clinical Nutrition, 75, 137–144. 10.1093/ajcn/75.1.137 11756071

[fsn3878-bib-0009] Colquhoun, A. , Arnold, M. , Ferlay, J. , Goodman, K. J. , Forman, D. , & Soerjomataram, I. (2015). Global patterns of cardia and non‐cardia gastric cancer incidence in 2012. Gut, 64, 1881–1888. 10.1136/gutjnl-2014-308915 25748648

[fsn3878-bib-0010] Costabile, A. , Klinder, A. , Fava, F. , Napolitano, A. , Fogliano, V. , Leonard, C. , … Tuohy, K. M. (2008). Whole‐grain wheat breakfast cereal has a prebiotic effect on the human gut microbiota: A double‐blind, placebo‐controlled, crossover study. The British Journal of Nutrition, 99, 110–120. 17761020 10.1017/S0007114507793923

[fsn3878-bib-0011] De Stefani, E. , Correa, P. , Boffetta, P. , Deneo‐Pellegrini, H. , Ronco, A. L. , & Mendilaharsu, M. (2004). Dietary patterns and risk of gastric cancer: A case‐control study in Uruguay. Gastric Cancer, 7, 211–220. 10.1007/s10120-004-0295-2 15616769

[fsn3878-bib-0012] Denova‐Gutierrez, E. , Hernandez‐Ramirez, R. U. , & Lopez‐Carrillo, L. (2014). Dietary patterns and gastric cancer risk in Mexico. Nutrition and Cancer, 66, 369–376. 10.1080/01635581.2014.884237 24628363

[fsn3878-bib-0013] Egeberg, R. , Olsen, A. , Christensen, J. , Johnsen, N. F. , Loft, S. , Overvad, K. , & Tjønneland, A. (2011). Intake of whole‐grain products and risk of prostate cancer among men in the Danish Diet, Cancer and Health cohort study. Cancer Causes & Control: CCC, 22, 1133–1139. 10.1007/s10552-011-9789-5 21656162

[fsn3878-bib-0014] Egeberg, R. , Olsen, A. , Loft, S. , Christensen, J. , Johnsen, N. F. , Overvad, K. , & Tjønneland, A. (2009). Intake of whole grain products and risk of breast cancer by hormone receptor status and histology among postmenopausal women. International Journal of Cancer, 124, 745–750. 10.1002/ijc.23992 19004010

[fsn3878-bib-0015] El‐Serag, H. B. , & Sonnenberg, A. (1999). Ethnic variations in the occurrence of gastroesophageal cancers. Journal of Clinical Gastroenterology, 28, 135–139. 10.1097/00004836-199903000-00010 10078821

[fsn3878-bib-0016] Ferlay, J. , Soerjomataram, I. , Dikshit, R. , Eser, S. , Mathers, C. , Rebelo, M. , … Bray, F. (2015). Cancer incidence and mortality worldwide: Sources, methods and major patterns in GLOBOCAN 2012. International Journal of Cancer, 136, E359–E386. 10.1002/ijc.29210 25220842

[fsn3878-bib-0017] Fraser, G. E. (1999). Associations between diet and cancer, ischemic heart disease, and all‐cause mortality in non‐Hispanic white California Seventh‐day Adventists. The American Journal of Clinical Nutrition, 70, 532s–538s. 10.1093/ajcn/70.3.532s 10479227

[fsn3878-bib-0018] Gil, A. , Ortega, R. M. , & Maldonado, J. (2011). Wholegrain cereals and bread: A duet of the Mediterranean diet for the prevention of chronic diseases. Public Health Nutrition, 14, 2316–2322. 10.1017/S1368980011002576 22166190

[fsn3878-bib-0019] Giovannucci, E. (1995). Insulin and colon cancer. Cancer Causes & Control: CCC, 6, 164–179. 10.1007/BF00052777 7749056

[fsn3878-bib-0020] Hakama, M. , & Saxen, E. A. (1967). Cereal consumption and gastric cancer. International Journal of Cancer, 2, 265–268. 10.1002/(ISSN)1097-0215 6041993

[fsn3878-bib-0021] Jacobs, D. R. Jr , Marquart, L. , Slavin, J. , & Kushi, L. H. (1998). Whole‐grain intake and cancer: An expanded review and meta‐analysis. Nutrition and Cancer, 30, 85–96. 10.1080/01635589809514647 9589426

[fsn3878-bib-0022] Jansen, M. C. , Bueno‐de‐Mesquita, H. B. , Rasanen, L. , Fidanza, F. , Menotti, A. , Nissinen, A. , … Kromhout, D. (1999). Consumption of plant foods and stomach cancer mortality in the seven countries study. Is grain consumption a risk factor? Seven Countries Study Research Group. Nutrition and Cancer, 34, 49–55. 10.1207/S15327914NC340107 10453441

[fsn3878-bib-0023] Karagulle, M. , Fidan, E. , Kavgaci, H. , & Ozdemir, F. (2014). The effects of environmental and dietary factors on the development of gastric cancer. Journal of B.U.ON., 19, 1076–1082. 25536619

[fsn3878-bib-0024] Kasum, C. M. , Jacobs, D. R. Jr , Nicodemus, K. , & Folsom, A. R. (2002). Dietary risk factors for upper aerodigestive tract cancers. International Journal of Cancer, 99, 267–272. 10.1002/(ISSN)1097-0215 11979443

[fsn3878-bib-0025] Kasum, C. M. , Nicodemus, K. , Harnack, L. J. , Jacobs, D. R. Jr , & Folsom, A. R. ; Iowa Women's Health Study . (2001). Whole grain intake and incident endometrial cancer: The Iowa Women's Health Study. Nutrition and Cancer, 39, 180–186. 10.1207/S15327914nc392_4 11759278

[fsn3878-bib-0026] Kono, S. , & Hirohata, T. (1996). Nutrition and stomach cancer. Cancer Causes & Control: CCC, 7, 41–55. 10.1007/BF00115637 8850434

[fsn3878-bib-0027] Kyro, C. , Olsen, A. , Landberg, R. , Skeie, G. , Loft, S. , Åman, P. , … Bueno‐de‐Mesquita, H. B. (2014). Plasma alkylresorcinols, biomarkers of whole‐grain wheat and rye intake, and incidence of colorectal cancer. Journal of the National Cancer Institute, 106, djt352. 24317181 10.1093/jnci/djt352 PMC3906988

[fsn3878-bib-0028] La Vecchia, C. , Negri, E. , Decarli, A. , D'Avanzo, B. , & Franceschi, S. (1987). A case‐control study of diet and gastric cancer in northern Italy. International Journal of Cancer, 40, 484–489. 10.1002/(ISSN)1097-0215 3117710

[fsn3878-bib-0029] Lafiandra, D. , Riccardi, G. , & Shewry, P. R. (2014). Improving cereal grain carbohydrates for diet and health. Journal of Cereal Science, 59, 312–326. 10.1016/j.jcs.2014.01.001 24966450 PMC4064937

[fsn3878-bib-0030] Lansdorp‐Vogelaar, I. , & Kuipers, E. J. (2016). Screening for gastric cancer in Western countries. Gut, 65, 543–544. 10.1136/gutjnl-2015-310356 26611232

[fsn3878-bib-0031] Larsson, S. C. , Giovannucci, E. , Bergkvist, L. , & Wolk, A. (2005). Whole grain consumption and risk of colorectal cancer: A population‐based cohort of 60,000 women. British Journal of Cancer, 92, 1803–1807. 10.1038/sj.bjc.6602543 15827552 PMC2362029

[fsn3878-bib-0032] Larsson, S. C. , Orsini, N. , & Wolk, A. (2006). Processed meat consumption and stomach cancer risk: A meta‐analysis. Journal of the National Cancer Institute, 98, 1078–1087. 10.1093/jnci/djj301 16882945

[fsn3878-bib-0033] Lei, Q. , Zheng, H. , Bi, J. , Wang, X. , Jiang, T. , Gao, X. , … Li, J. (2016). Whole grain intake reduces pancreatic cancer risk: A meta‐analysis of observational studies. Medicine, 95, e2747 10.1097/MD.0000000000002747 26945361 PMC4782845

[fsn3878-bib-0034] Levi, F. , Pasche, C. , Lucchini, F. , Chatenoud, L. , Jacobs, D. R. Jr , & La Vecchia, C. (2000). Refined and whole grain cereals and the risk of oral, oesophageal and laryngeal cancer. European Journal of Clinical Nutrition, 54, 487–489. 10.1038/sj.ejcn.1601043 10878650

[fsn3878-bib-0035] Lissowska, J. , Gail, M. H. , Pee, D. , Groves, F. D. , Sobin, L. H. , Nasierowska‐Guttmejer, A. , … Chow, W. H. (2004). Diet and stomach cancer risk in Warsaw, Poland. Nutrition and Cancer, 48, 149–159. 10.1207/s15327914nc4802_4 15231449

[fsn3878-bib-0036] Lucenteforte, E. , Scita, V. , Bosetti, C. , Bertuccio, P. , Negri, E. , & La Vecchia, C. (2008). Food groups and alcoholic beverages and the risk of stomach cancer: A case‐control study in Italy. Nutrition and Cancer, 60, 577–584. 10.1080/01635580802054512 18791920

[fsn3878-bib-0037] Lunet, N. , Valbuena, C. , Vieira, A. L. , Lopes, C. , Lopes, C. , David, L. , … Barros, H. (2007). Fruit and vegetable consumption and gastric cancer by location and histological type: Case–control and meta‐analysis. European Journal of Cancer Prevention, 16, 312–327. 10.1097/01.cej.0000236255.95769.22 17554204

[fsn3878-bib-0038] Malvezzi, M. , Bonifazi, M. , Bertuccio, P. , Levi, F. , La Vecchia, C. , Decarli, A. , & Negri, E. (2010). An age‐period‐cohort analysis of gastric cancer mortality from 1950 to 2007 in Europe. Annals of Epidemiology, 20, 898–905. 10.1016/j.annepidem.2010.08.013 21074104

[fsn3878-bib-0039] McCullough, M. L. , Robertson, A. S. , Jacobs, E. J. , Chao, A. , Calle, E. E. , & Thun, M. J. (2001). A prospective study of diet and stomach cancer mortality in United States men and women. Cancer Epidemiology, Biomarkers & Prevention, 10, 1201–1205. 11700269

[fsn3878-bib-0040] Modan, B. , Lubin, F. , Barell, V. , Greenberg, R. A. , Modan, M. , & Graham, S. (1974). The role of starches in etiology of gastric cancer. Cancer, 34, 2087–2092. 10.1002/(ISSN)1097-0142 4434338

[fsn3878-bib-0041] Plummer, M. , Franceschi, S. , Vignat, J. , Forman, D. , & de Martel, C. (2015). Global burden of gastric cancer attributable to *Helicobacter pylori* . International Journal of Cancer, 136, 487–490. 10.1002/ijc.28999 24889903

[fsn3878-bib-0042] Ramón, J. M. , Serra, L. , Cerdó, C. , & Oromí, J. (1993). Dietary factors and gastric cancer risk a case‐control study in Spain. Cancer, 71, 5. 8448737 10.1002/1097-0142(19930301)71:5<1731::aid-cncr2820710505>3.0.co;2-x

[fsn3878-bib-0043] Research, W. C. R. F. A. I. f. C. (2007), AICR, Washington,DC .

[fsn3878-bib-0044] Roager, H. M. , Vogt, J. K. , Kristensen, M. , Hansen, L. B. S. , Ibrügger, S. , & Mærkedahl, R. B. (2017). Whole grain‐rich diet reduces body weight and systemic low‐grade inflammation without inducing major changes of the gut microbiome: A randomised cross‐over trial. Gut, [Epub ahead of print]. 10.1136/gutjnl-2017-314786 PMC6839833 29097438

[fsn3878-bib-0045] Rota, M. , Pelucchi, C. , Bertuccio, P. , Matsuo, K. , Zhang, Z. F. , Ito, H. , … Vecchia, C. (2017). Alcohol consumption and gastric cancer risk‐A pooled analysis within the StoP project consortium. International Journal of Cancer, 141, 1950–1962. 10.1002/ijc.30891 28718913

[fsn3878-bib-0046] Schatzkin, A. , Mouw, T. , Park, Y. , Subar, A. F. , Kipnis, V. , Hollenbeck, A. , … Thompson, F. E. (2007). Dietary fiber and whole‐grain consumption in relation to colorectal cancer in the NIH‐AARP Diet and Health Study. The American Journal of Clinical Nutrition, 85, 1353–1360. 10.1093/ajcn/85.5.1353 17490973

[fsn3878-bib-0047] Schatzkin, A. , Park, Y. , Leitzmann, M. F. , Hollenbeck, A. R. , & Cross, A. J. (2008). Prospective study of dietary fiber, whole grain foods, and small intestinal cancer. Gastroenterology, 135, 1163–1167. 10.1053/j.gastro.2008.07.015 18727930 PMC3513331

[fsn3878-bib-0048] Shamberger, R. J. , Tytko, S. , & Willis, C. E. (1972). Antioxidants in cereals and in food preservatives and declining gastric cancer mortality. Cleveland Clinic Quarterly, 39, 119–124. 10.3949/ccjm.39.3.119 5084377

[fsn3878-bib-0049] So, W. K. , Law, B. M. , Law, P. T. , Chan, C. W. , & Chair, S. Y. (2016). Current hypothesis for the relationship between dietary rice bran intake, the intestinal microbiota and colorectal cancer prevention. Nutrients, 8, 569 10.3390/nu8090569 PMC5037554 27649240

[fsn3878-bib-0050] Steevens, J. , Schouten, L. J. , Goldbohm, R. A. , & van den Brandt, P. A. (2010). Alcohol consumption, cigarette smoking and risk of subtypes of oesophageal and gastric cancer: A prospective cohort study. Gut, 59, 39–48. 10.1136/gut.2009.191080 19828467

[fsn3878-bib-0051] Stephen, A. M. (1994). Whole grains–impact of consuming whole grains on physiological effects of dietary fiber and starch. Critical Reviews in Food Science and Nutrition, 34, 499–511. 10.1080/10408399409527677 7811380

[fsn3878-bib-0052] Sturgess, R. P. , Ellis, H. J. , & Ciclitira, P. J. (1991). Cereal chemistry, molecular biology, and toxicity in coeliac disease. Gut, 32, 1055–1060. 10.1136/gut.32.9.1055 1916491 PMC1379050

[fsn3878-bib-0053] Terry, P. , Lagergren, J. , Ye, W. , Wolk, A. , & Nyren, O. (2001). Inverse association between intake of cereal fiber and risk of gastric cardia cancer. Gastroenterology, 120, 387–391. 10.1053/gast.2001.21171 11159879

[fsn3878-bib-0054] Tetens, I. (2017). Substituting whole grain for refined grain: What is needed to strengthen the scientific evidence for health outcomes? The American Journal of Clinical Nutrition, 105, 545–546. 10.3945/ajcn.117.152496 28202476 PMC5320419

[fsn3878-bib-0055] Torre, L. A. , Bray, F. , Siegel, R. L. , Ferlay, J. , Lortet‐Tieulent, J. , & Jemal, A. (2015). Global cancer statistics, 2012. CA: A Cancer Journal for Clinicians, 65, 87–108. 25651787 10.3322/caac.21262

[fsn3878-bib-0056] Vanamala, J. K. P. , Massey, A. R. , Pinnamaneni, S. R. , Reddivari, L. , & Reardon, K. F. (2017). Grain and sweet sorghum (*Sorghum bicolor* L. Moench) serves as a novel source of bioactive compounds for human health. Critical Reviews in Food Science and Nutrition, 29, 1–15. 10.1080/10408398.2017.1344186 28662339

[fsn3878-bib-0057] Wang, J. B. , Fan, J. H. , Dawsey, S. M. , Sinha, R. , Freedman, N. D. , Taylor, P. R. , … Abnet, C. C. (2016). Dietary components and risk of total, cancer and cardiovascular disease mortality in the Linxian Nutrition Intervention Trials cohort in China. Scientific Reports, 6, 22619 10.1038/srep22619 26939909 PMC4778051

[fsn3878-bib-0058] Wang, X. Q. , Yan, H. , Terry, P. D. , Wang, J. S. , Cheng, L. , Wu, W. A. , & Hu, S. K. (2012). Interaction between dietary factors and *Helicobacter pylori* infection in noncardia gastric cancer: A population‐based case‐control study in China. Journal of the American College of Nutrition, 31, 10. 10.1080/07315724.2012.10720447 23529995

[fsn3878-bib-0059] Ward, M. H. (1999). Dietary factors and the risk of gastric cancer in Mexico City. American Journal of Epidemiology, 149, 8. 10.1093/oxfordjournals.aje.a009736 10342801

[fsn3878-bib-0060] Wong, B. C. , Lam, S. K. , Wong, W. M. , Chen, J. S. , Zheng, T. T. , Feng, R. E. , & Lai, K. C. … for the China Gastric Cancer Study Group . (2004). Helicobacter pylori eradication to prevent gastric cancer in a high‐risk region of China: A randomized controlled trial. JAMA, 291, 187–194. 10.1001/jama.291.2.187 14722144

[fsn3878-bib-0061] Zhang, Z. F. , Kurtz, R. C. , Yu, G. P. , Sun, M. , Gargon, N. , Karpeh, M. Jr , … Harlap, S. (1997). Adenocarcinomas of the esophagus and gastric cardia: The role of diet. Nutrition and Cancer, 27, 298–309. 10.1080/01635589709514541 9101561

[fsn3878-bib-0062] Zhang, Z. , Xu, G. , Ma, M. , Yang, J. , & Liu, X. (2013). Dietary fiber intake reduces risk for gastric cancer: A meta‐analysis. Gastroenterology, 145(113–120), e113 10.1053/j.gastro.2013.04.001 23567349

[fsn3878-bib-0063] Zhou, Y. , Zhuang, W. , Hu, W. , Liu, G. J. , Wu, T. X. , & Wu, X. T. (2011). Consumption of large amounts of Allium vegetables reduces risk for gastric cancer in a meta‐analysis. Gastroenterology, 141, 80–89. 10.1053/j.gastro.2011.03.057 21473867

